# Large-Area Monocrystalline Copper Microflake Synthesis

**DOI:** 10.1021/acs.jpcc.5c00654

**Published:** 2025-06-16

**Authors:** Elif Nur Dayi, Diotime Pellet, Priscila Vensaus, Fatemeh Kiani, Alan R. Bowman, Omer Can Karaman, Giulia Tagliabue

**Affiliations:** Laboratory of Nanoscience for Energy Technologies (LNET), STI, 27218 École Polytechnique Fédérale de Lausanne, 1015 Lausanne, Switzerland

## Abstract

Copper is one of the most extensively studied materials for energy conversion and catalytic systems, with a wide range of other applications, from nanophotonics to biotechnology. However, existing synthesis methods are limited with many undesirable byproducts and poorly defined morphologies. Here, we report an on-substrate wet synthesis approach that yields purely metallic and monocrystalline Cu microflakes with an exposed (111) crystalline surface. By systematically studying the growth mechanism, we achieve unprecedented sizes of more than 130 μm, which is 2 orders of magnitude larger than reported in most previous studies, along with high aspect ratios of over 400. Furthermore, we show significantly higher stability against oxidation provided by the halide adlayer, which also eliminates the need for any organic surfactants in the synthesis. Overall, our facile synthesis approach delivers an exciting avenue for the emerging fields of catalysis and nanophotonics.

## Graphical abstract

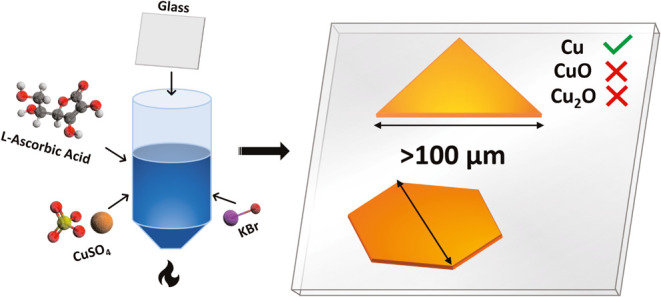

## Introduction

The controlled growth of metallic nanomaterials with tailored morphology, high purity, and well-defined crystallinity is a long-standing challenge with crucial implications for many applications in plasmonics and nanophotonics,^1–3^ optoelectronics,^4,5^ flexible electronics,^6^ energy conversion, and catalysis.^1,7–9^ In particular, copper has emerged as a key material for energy applications: it is the most extensively studied catalyst for the CO_2_ reduction reaction (CO_2_RR), thanks to its rare combination of Earth-abundance, low cost, and unique ability to sustain the formation of carbon–carbon bonds on its surface and ultimately the generation of high energy-density multicarbon products.^10–12^

Catalytic, thermal, and optoelectronic properties of Cu nanomaterials depend on the preparation technique and material quality.^13–15^ Although there is a plethora of methods for the growth of Cu nanoparticles in the literature, such as solvothermal,^16^ microwave-assisted growth,^17^ electrochemical reduction^18^ and wet synthesis,^8,19^ a method that is simultaneously simple, scalable, and cost-effective while enabling controlled growth of large-aspect-ratio Cu nanomaterials with smooth, exposed (111) surfaces remains elusive. This specific surface orientation can be beneficial for a wide range of applications, such as directing the chemical selectivity of reactions^8,13^ or achieving unique plasmonic properties.^20^

Table 1 gives an overview of the state of the art in the synthesis of thin Cu nanomaterials with (111) orientation, namely, nanoflakes, nanoplates, and nanosheets. However, these methods offer either limited lateral size, low yield, or poor selectivity toward a specific geometry. To the best of our knowledge, most reported Cu flakes have a lateral size of only a few microns.^8,21^ In the few cases where the flakes are larger than 5 μm, they are not well separated from each other and are surrounded by undesired side products such as nanorods.^6,8,22^ In addition, these methods utilize various surfactants and organic molecules, including glucose, hexadecyltrimethylammonium bromide (CTAB), and hexamethylenetetramine (HMTA).^8,23^ These organic surfactants, which adhere to the surface, can reduce the available active sites, restrict access to the surface, may interfere with chemical reactions and lead to misinterpretations of catalytic activity and selectivity.^24^ Removal of these molecules from the surface requires additional treatment, such as annealing or acid baths, which impairs the surface quality. A surfactant-free method can be advantageous for avoiding these challenges.

**Table 1:** Summary of Reported Syntheses for Thin Cu Materials with a (111) Surface Orientation

material	method	reactants	lateral size	aspect ratio	refs
Cu nanoplatelets	electroreduction	Cu(CN)_2_^–^	≈200 nm	NA	^25^
Cu nanoplatelets	wet synthesis	CuBr	≈1.019 ± 0.519 μm	NA	^19^
		DPP			
		OLAM			
Cu nanosheets	wet synthesis	Cu(NO_3_)_2_	≈1.7 ± 0.5 μm	340	^8^
		l-ascorbic acid			
		HMTA			
		CTAB			
Cu nanosheets	wet synthesis	Cu(NO_3_)_2_	≈1.7 ± 0.5 μm	300	^23^
		l-ascorbic acid			
		HMTA			
		TTAB			
Cu nanoplates	wet synthesis	CuSO_4_·5H_2_O	≈3.4 μm	NA	^26^
		l-ascorbic acid			
		CTAB			
Cu nanoplatelets	wet synthesis	CuCl_2_·2H_2_O	≈5 μm	300	^6^
		d-glucose			
		HDA			
		NaI			
Cu nanoplatelets	wet synthesis	CuBr_2_	≈8.03 ± 3.18 μm	22	^22^
		l-ascorbic acid			
		BPEI			
Cu nanoplates	wet synthesis	CuBr_2_	≈10.97 ± 3.45 μm	NA	^27^
		l-ascorbic acid			
		BPEI			
		Ag			
Cu microflakes	substrate-assisted wet synthesis	CuSO_4_·5H_2_O	>130 μm	400	this work
		l-ascorbic acid			
		KBr			

In this study, we present a straightforward route for surfactant-free growth of monocrystalline Cu microflakes and address the major bottlenecks in existing methods, such as small size, low selectivity, reduced yield, and poorly defined morphologies. Our optimized recipe is constructed by carefully monitoring the effect of various parameters on the growth process, such as the reaction temperature, salt precursors, halide choice, and concentration. This allows us to push the lateral dimensions from just a few micrometers to over a hundred. We combine these efforts with advanced characterization techniques to establish the monocrystallinity, (111) orientation, and elemental composition of the flakes and gain insight into the growth mechanism. Remarkably, we show that thanks to the halide adlayer, the flakes exhibit significantly extended stability against surface oxidation, a phenomenon that challenges applications of Cu-based nanomaterials. Lastly, while most flakes exhibit a smooth basal surface, at elevated temperatures in particular, we observe the emergence of distinct surface features, such as atomic steps, which are particularly valuable for probing surface-dependent phenomena. These Cu microflakes with tunable morphology will enable studies of the optoelectronic properties of monocrystalline metals as well as of energy and chemical conversion processes in previously unprecedented detail.

## Methods

### Synthesis

An experimental setup previously presented by Kiani and co-workers^28^ was used. All chemical reagents were analytical grade and were used as purchased from Sigma-Aldrich. First, selected concentrations of the salt precursor CuSO_4_·5H_2_O and l-ascorbic acid (10 mM CuSO_4_·5H_2_O and 30 mM l-ascorbic acid for the standard recipe) were added to 20 mL of ultrapure water in a standard 50 mL polypropylene Falcon tube used as a reactor. Depending on the experiment, halides such as KBr, KI, or KCl were added to the same solution in various concentrations (4.2 mM for the standard recipe) to serve as a capping and structure-directing agent. The final aqueous solution was then stirred vigorously for 30–60 min at room temperature.

To prepare the substrate, two borosilicate glass substrates (24 mm × 24 mm, #1.5, Epredia) were cleaned by sonication in acetone and deionized water for 10 min each and dried by nitrogen blowing. The clean substrates were individually immersed in an aqueous growth solution, which was previously stirred homogeneously. The stirring was then stopped, and substrates were placed in the tube with an upward tilted orientation so that they were immobilized during growth. The tube was sealed, placed in a beaker filled with water, and covered with aluminum foil to avoid possible light-induced effects. The system was then heated to the desired temperature (80 °C, unless otherwise specified) at a ramp rate of 2 °C/min on a hot plate without stirring. At the end of the growth window, heating was stopped, and the substrates were immediately and individually removed. They were thoroughly rinsed with ethanol and deionized water, three times each, followed by drying with nitrogen blowing in order to remove all unreacted species . The samples were stored either in an inert atmosphere or under ambient conditions for stability tests.

### Materials Characterization

Optical micrographs were recorded after each experiment across different regions of the samples with a Nikon inverted optical microscope (Nikon Ti2A) and analyzed with commercial software ImageJ (Ver.1.8.0). Lateral size was measured by edge length for triangular flakes and by diagonal length for hexagonal and truncated flakes to ensure consistency with previous studies.^29,30^ Experiments for parametric studies were repeated at least 3 times each. The analysis was restricted to flakes that are larger than 5 μm, and flakes with any surface deformation were not considered. For the statistical analysis of yield, optical micrographs were converted to 32-bit binary images, followed by a color thresholding and the particle analysis function to quantify the area. For each formulation, several images were analyzed to achieve a total area of up to 1 mm^2^. Particles with an area of less than 0.5 μm^2^ were not included in the analysis to prevent outlier pixels from affecting the analysis.

The X-ray diffraction experiments of regular theta–theta scans were performed on a Panalytical Empyrean X-ray polycrystalline diffractometer in Bragg–Brentano geometry, equipped with a long-focused sealed Cu X-ray tube (λKα = 1.5418 Å) and PIXcel 1D X-ray detector. The patterns were collected in continuous mode between 10 and 100° (2θ), with a step-size of 0.02626° over 19 h. Background subtraction and peak identification were performed with HighScore plus v4.9 and PDF5+ v2024.^31^ The microscopic absorptance measurements were performed via the methodology described in detail by Bowman et al.,^32^ where experimental results were benchmarked against transfer matrix method calculations on Au. The measurements were recorded by using a microscope (Nikon Eclipse T2) coupled to a spectrometer (Princeton Instruments Spectra Pro HRS-500) and a laser-driven white light source output (Energetica LDLS) through a fiber with a 100 μm core diameter. For transmission, incident light was collimated through a condenser lens and a bare glass substrate was used as a reference. For reflection, incident light was focused on the center of the back focal plane of a 60× objective (Nikon S Plan Fluor ELWD, NA = 0.7) and a mirror with known spectral response (Thorlabs PF10–03-P10–10) was used as a reference. The reflection and transmission spectra were measured for a single flake at a time for multiple samples, and the data was analyzed in MATLAB to acquire the absorptance spectrum.

Scanning electron microscopic energy dispersive X-ray spectroscopy (SEM-EDS) was performed by using a ZEISS Merlin field emission microscope with an accelerating voltage of 15 kV and a working distance of 8.5 mm. Before the measurements, the insulating sample was coated with a Au (15 nm) or C (12 nm) layer by sputtering to achieve an electrical conductivity. Atomic force microscopy (AFM) images and height profiles were acquired using a Bruker Fast-Scan AFM in ScanAsyst mode, and the data was processed with Gwyddion commercial software. TEM cross-sectional analyses were performed on a double aberration corrected FEI Titan Themis at 300 kV in the [110] crystal direction of the Cu flake. The TEM lamella was fabricated from a Cu flake grown on the substrate with a dual-beam focused ion beam (FIB)/SEM (Zeiss NVision 40). Prior to FIB milling, the sample was coated with a thin (ca. 20 nm) Au layer to ensure electrical conductivity, and the region of interest was finally protected by FIB-assisted carbon deposition (ca. 1 μm). The prepared fused silica/Cu(111)/Au/C stacked structure was extracted by FIB milling (30 kV Ga+ beam), fixed on a Mo-TEM grid, and stored under vacuum before measurements to prevent oxidation. Avogadro software^33^ was used to draw the molecular structures included in the Table of Contents.

## Results and Discussion

While the synthesis of large-area Au monocrystalline flakes is well established in the literature, their Cu counterparts have not been successfully reported despite having high potential for applications in energy and photonics. Here, we present a method for growing large-area Cu microflakes on a substrate. Adapting methods used for Au to Cu is not trivial, as the two materials differ significantly, e.g., in terms of oxidation behavior and lattice parameters. Thus, we explored a wide range of chemical components and growth conditions to find a combination that fosters Cu microflake growth.

Our synthesis configuration consists of a polypropylene tube that acts as a reactor, two borosilicate glass substrates placed inside the tube in a tilted orientation, a temperature controller, and a heating plate, similar to the system previously reported herein.^28^ The reaction medium is ultrapure water (20 mL), in which the respective precursors are dissolved. The proposed halide-assisted wet-synthesis on glass substrates offers the following major advantages: The shaping and complexation effects of the halides can favor lateral anisotropic growth of the flakes, while the growth of nanoparticles on the substrate brings higher quality as aggregation, bending, and contamination by small particles are avoided, in contrast to methods such as dropcasting after synthesis.

Nanoparticle formation can generally be divided into two phases: the initial nucleation phase, where individual atoms coalesce to form clusters or seeds, and the subsequent growth phase, during which these seeds develop into crystals with specific shapes. The growth of nanomaterials with a desired crystalline orientation can be achieved through the use of capping agents whose binding affinity can promote or hinder the growth of a particular facet and control the overall morphology of the particles.

With the goal of understanding the merits enabled by the on-substrate growth on Cu flake growth, we first adapted a recipe from literature by Luc et al.,^8^ where Cu­(NO_3_)_2_·3H_2_O precursor and l-ascorbic acid reducing agent are combined with hexadecyltrimethylammonium bromide (CTAB) and hexamethylenetetramine (HMTA), which act as organic surfactants at 80 °C for 3 h. This recipe produced flakes with an average lateral size of ≈5.5 ± 1.8 μm, over three times larger than the reported ≈1.7 ± 0.5 μm, highlighting the advantage of the on-substrate approach over colloidal synthesis. We extended the reaction time from 3 to 20 h aimed to increase particle count for statistical analysis (Figure S1). However, while the average size increased, flakes were often covered by byproducts, with limited selectivity for triangular and hexagonal shapes, indicative of (111) surface orientation. Repeated syntheses showed significant variations in flake size and product distribution, likely due to the rapid degradation of organic compounds. Additionally, surface coverage by long-chain organic ligands remained a concern for catalytic applications.

To address issues related to surface attachment and degradation of organic surfactants, we opted for an inorganic ligand instead. Studies on halide-assisted Cu and metal growth show that halide ions (Br^–^, Cl^–^) selectively adsorb on the {111} basal plane, enabling surface passivation.^22,28,34,35^ We first tested potassium bromide (KBr, 10 mg, 4.2 mM) due to its ionic radius being close to that of Cu, potentially reducing lattice strain and improving structural compatibility. To maintain a slow reduction rate for thin Cu flake formation, we retained l-ascorbic acid (30 mM) as a mild reducing agent.^36^ KBr reduced byproducts (Figure S2a); however, the flake size and yield remained limited. Further optimization of halide ions and a quantitative analysis of yield and selectivity will be presented later.

To find an effective combination that can promote the average size and yield of the flakes, we added a fixed amount of KBr (10 mg, 4.2 mM) and tested several Cu salts with different anions, such as SO_4_^2–^, NO_3_^–^, Cl^–^, and CH_3_COO^–^, as the chemical affinities of the anions are known to be crucial for the crystallization and growth mechanisms.^37^ The results of this study are summarized in Figure S2, where a significant difference can be seen in the products between the different precursors, leading us to choose CuSO_4_ as the salt precursor.

This halide-assisted recipe, in which KBr is combined with CuSO_4_ at 80 °C for 20 h, was the first combination to consistently generate flakes >10 μm growing on both sides of the substrates. Therefore, we refer to it as the standard recipe and use it as the basis for optimization studies and to have comparability among different characterization approaches. In this synthesis, the flakes also grow in the colloidal solution; however, their surface quality and selectivity are poor compared with growth on the substrate. A comparison can be seen in Figure S3, where the particles grown in the solution using the drop-casting method are contrasted with the particles grown on the substrate. The former shows structural deformation of the flakes and a variety of byproducts, such as clusters. Therefore, for the remainder of the study, we focus our analysis on the on-substrate grown flakes.

Figure 1 represents the Cu flake growth approach discussed in this paper and demonstrates the strong influence of halide ions on the control of the Cu nanoflake lateral size and final morphology. Figure 1b shows that the lateral dimension doubles when KBr is chosen as the halide ion compared to other halides, such as KI and KCl or the case without halide. To better understand the exact role of KBr in the growth mechanism, we varied its concentration, as shown in Figure 1c. Up to 5 mM, the average flake size is proportional to the KBr concentration. However, at concentrations higher than 5 mM, we observed significant changes in flake morphology due to complexation and oxidative etching effects, which also increase with higher concentration.^38^ The effect of the high KBr content is visible on the scanning electron microscopy-energy dispersive X-ray spectroscopy (SEM-EDS) elemental maps (see Figure S4a), where the flakes become visibly thinner in the center. Since precise tuning of the KBr concentration is important to ensure a smooth surface while supporting lateral growth, we chose the optimal KBr concentration as 4.2 mM.

**Figure 1:**
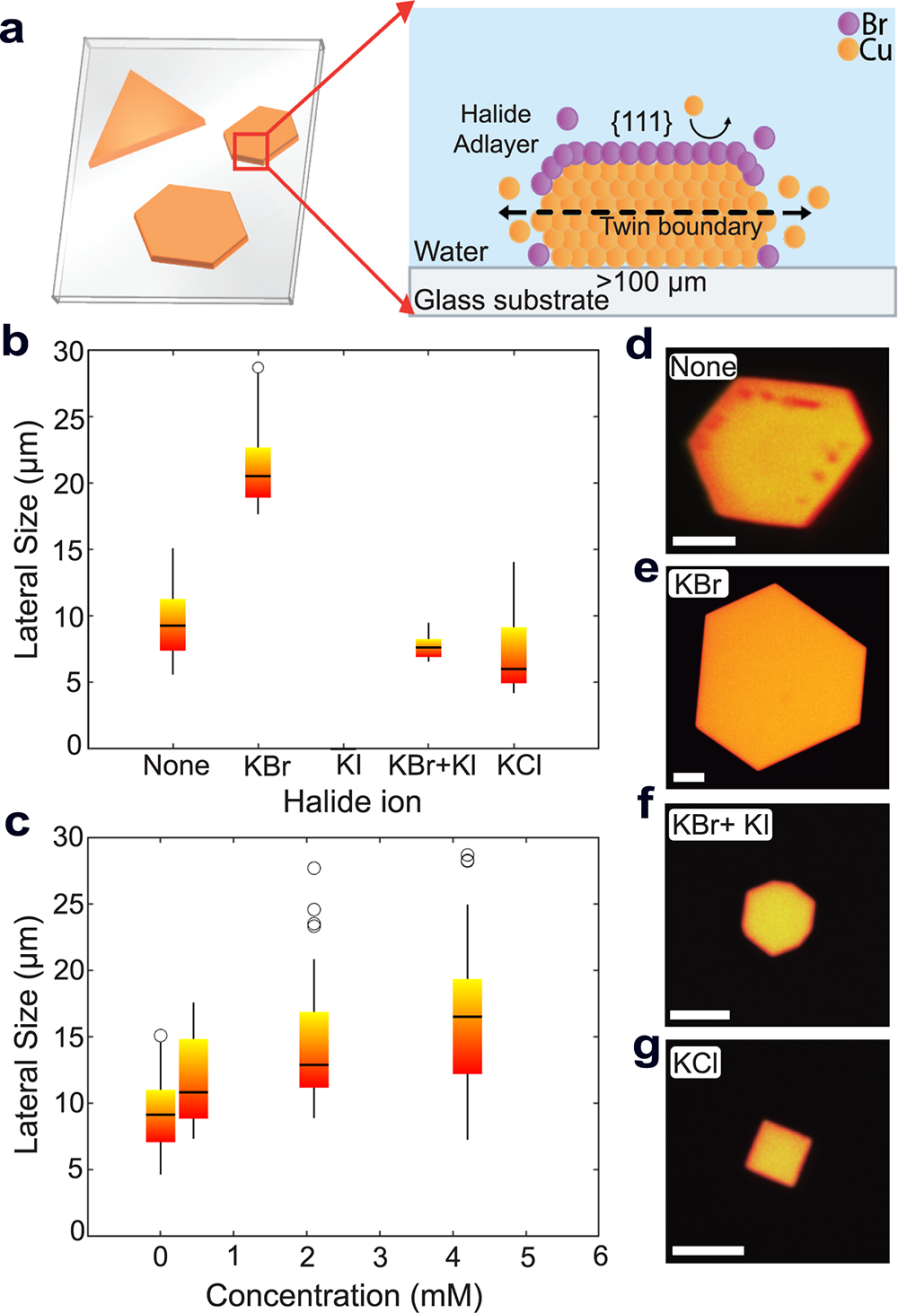
Overview of the Cu microflake growth on the substrate and the halide-controlled morphology. (a) Illustration of the Cu flakes grown on substrate, planar (left) and cross-sectional (right) views. The halide adlayer (purple) promotes growth in the lateral direction. (b) Average lateral size obtained using different halide ions. Resulting morphology for each condition is shown in (c–f), with an exception for the use of KI, for which there was no visible formation of flakes. The scale bar represents 5 μm. (c) Dependence of flake lateral size on KBr concentration. (d) Anisotropic flakes with surface impurities were observed when no halide ions are used. (e) Hexagonal flake obtained when using 4.2 mM KBr. (f) Etched flakes when KBr and KI are included simultaneously at equal concentrations of 4.2 mM. (g) Square-like flakes formed when 4.2 mM KCl is used.

Apart from the size, we also observe significant changes in the facets depending on the halide ion. For example, while it is possible to grow flakes up to 10 μm without the halides, the facet growth is less controlled, resulting in a nonequilateral, elongated hexagonal shape, as shown in Figure 1d. When KBr is present as a shape-directing agent, we instead observe equilateral triangles and hexagons, which are signs of isotropic lateral growth. In addition, flakes grown without halide tend to show dark spots on the surface of bright-light micrographs immediately after synthesis, which could be caused by partial oxidation. These dark spots are not observed in KBr-assisted growth shown in Figure 1e, suggesting that the halide ions not only direct the shape but also act as a capping agent and protect the surface from oxidation, consistent with previous reports.^34^ No significant flake growth was observed when using KI and the combination of KBr with KI resulted in smaller flakes with a larger number of smaller side facets, as visible in Figure 1f, likely due to strong complexation effects. The flake size was further reduced when only KCl was used, with a significant change in surface orientation, as shown in Figure 1g.

Upon investigating the role of halide ions, we employed numerous advanced characterization techniques to evaluate the stability, surface quality, crystallinity, and metallic nature of the flakes. Unless specified otherwise, all measurements were performed on Cu flake samples grown on a glass substrate by using the standard recipe introduced earlier in the text.

The X-ray diffraction (XRD) pattern shown in Figure 2a, exhibits two prominent peaks. The strong diffraction peak at 43.5° confirms that the Cu flakes are monocrystalline with {111} basal planes. Meanwhile, the smaller peak at 27.4° indicates {111} orientation for CuBr.^39^ EDS spectra of flakes grown using different concentrations of KBr, shown in Figure 2b, suggest that the Br signal from a flake prepared with the standard recipe (4.2 mM KBr, green) is near the detection limit and should not produce a strong peak in the XRD measurements. For flakes grown with a higher Br content of 10 mM (pink), a continuous bromide layer forms on the surface, as observed in Figure S4a. Additionally, EDS analyses of crystallites confirm the presence of CuBr particles on the glass surface, clarifying the origin of this peak (Figure S4b). Based on these results, we attribute this peak to a convolution of the signal from the halide layer and the crystallites. It is noteworthy that the XRD measurements were conducted one month after synthesis. Yet, no additional peaks corresponding to CuO or Cu_2_O were observed, highlighting the pivotal role of bromide ligands in mitigating surface oxidation and extending flake stability. Moreover, bright-field micrographs recorded from a flake immediately after synthesis and after 3 weeks under atmospheric conditions show that the flakes remain well-preserved (see Figure S4c,d), consistent with the XRD results.

**Figure 2:**
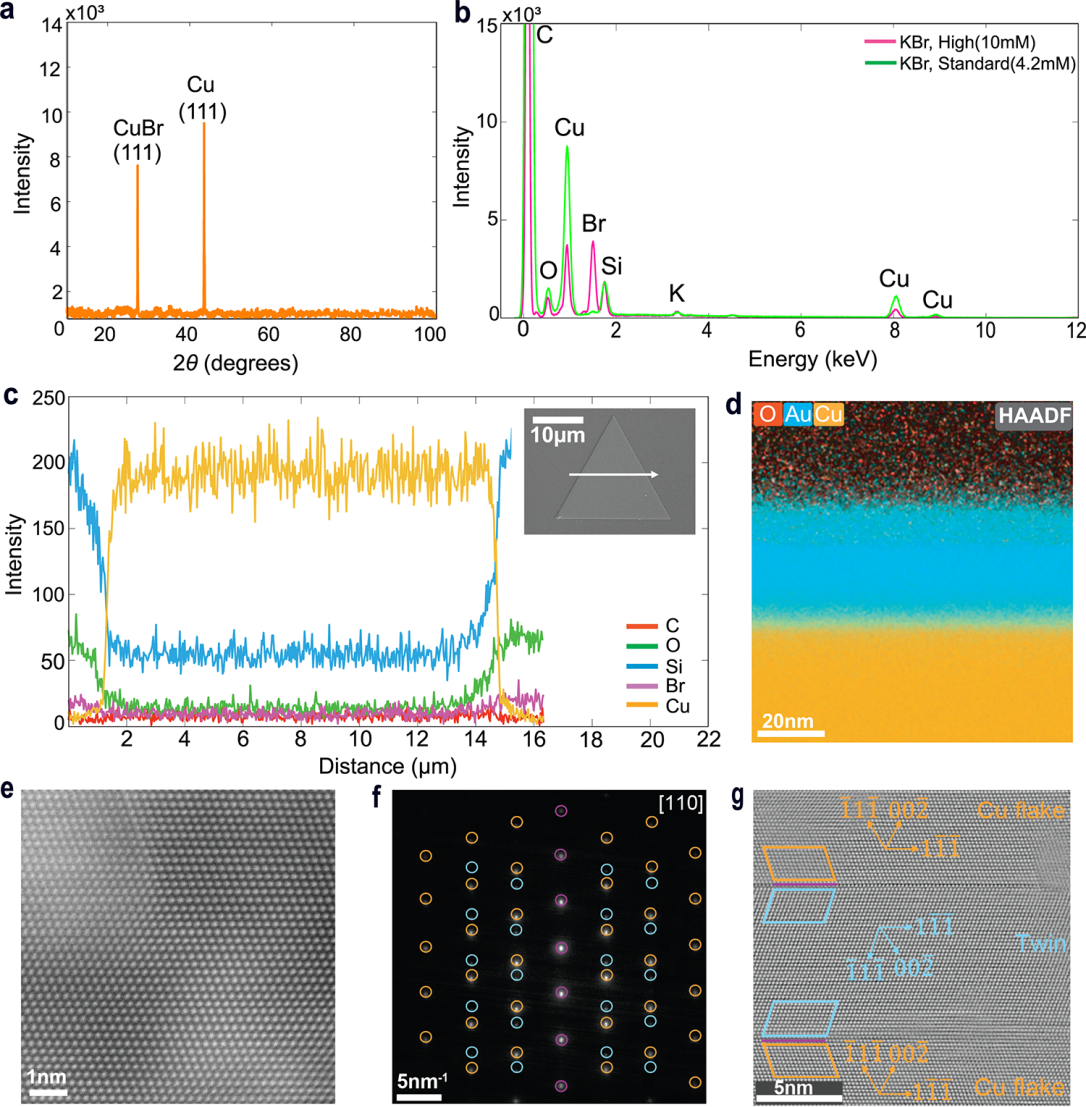
Compositional analysis of the Cu flakes. (a) XRD pattern of Cu flakes grown on a glass substrate. (b) EDS spectra were taken from an area inside the flake region for two samples prepared using different KBr concentrations: high (10 mM, pink) and standard (4.2 mM green). The samples were coated with Au for electrical conductivity. The Si and O signal is introduced by the glass substrate underneath as seen in part (c). (c) EDS line scans for C­(red), O­(green), Si­(blue), Br­(pink), and Cu­(orange) recorded for the flake shown in the inset, along the direction indicated by the white arrow. (d) HAADF-STEM image of the cross-sectional Cu flake (orange) TEM lamella with conductive Au layer­(blue) on top. The lack of an O signal­(red) on the flake region confirms the metallic nature of the flake. (e) Atomically resolved HR-TEM image of the Cu flake. (f) SAED pattern taken along the [110] direction showing a 6-fold symmetric fcc-Cu system along with distinct reflections from the nearby twinned region: orange circles mark reflections of the main Cu flake, blue circles mark reflections of the twin, and purple circles mark common reflections at the twin boundary. (g) HR-TEM image of the twin region analyzed in panel (f).

Figure 2c shows the elemental EDS line scans performed on the Cu flake included in the inset along the direction indicated by the arrow. A strong signal of Cu on the flake is visible, while the signal of the O drops inside the flake and increases back on the substrate region, further confirming the metallic nature of the flakes. Subsequently, we performed transmission electron microscopy (TEM) measurements on a cross-sectional Cu flake lamella prepared by focused ion beam-scanning electron microscopy (FIB-SEM, see Methods section). The insulating sample was sputter-coated with Au and C to improve the electrical conductivity. Figure 2d shows the High-Angle Annular Dark-Field scanning transmission electron microscopy (HAADF-STEM) image in the cross section, where O (red) is not observed in the Cu region (orange). We coupled this measurement with electron energy loss spectroscopy (EELS), where the O signal from the flake region is at noise levels (Figure S5). These analyses are in accordance with the SEM-EDS and XRD analyses and verify the metallic nature of the Cu flakes. Moreover, microscale absorptance measurements on the flakes were compared with the theoretical response of Cu of a similar thickness range (100–300 nm) calculated by the transfer matrix method using previously reported optical paramaters for Cu,^40^ for which the details are provided under Methods section. The strong agreement between the experiment and the theoretical optical response further proves the metallic nature of the flakes (Figure S6).

Atomically resolved high-resolution TEM images (HRTEM) in Figure 2e unveil the monocrystalline structure. The selective area diffraction (SAED) pattern in Figure 2f was recorded from a nearby region along the [110] direction. The indexation performed with CrysTBox software unveils that the reflections match the fcc-Cu system.^41^ The SAED pattern also reveals the presence of a twin boundary that extends parallel to the basal plane of the flake and mirrors the {111} lattice planes of the fcc.

The crucial role of stacking faults such as twin defects to sustain anisotropic growth of thin materials was introduced by Lofton and Sigmund and confirmed in several studies.^28,42,43^ Twin planes provide side facets for the attachment of Cu adatoms, and multiple twinning allows the side facets to regenerate each other. In addition, the energy barrier for addition of atoms in the vertical direction is increased, all together allowing realization of larger lateral sizes and higher aspect ratios. As expected from this growth mechanism, we observe a twin defect along the flake that can favor the lateral growth, for which the SAED pattern and HRTEM images are shown in Figure 2e,f, respectively. Reflections from twinned regions are enclosed in blue, reflections from the Cu flake are in orange, and last reflections from the twin mirror plane are marked in purple.

Following the investigations on structural and chemical composition, we proceed to optimize the maximum lateral size by monitoring the effects of various factors such as the growth period, reaction temperature, and salt precursor concentration on the growth mechanism. For these experiments, the standard recipe, which refers to 80 °C, 20 h reaction time, 10 mM CuSO_4_·5H_2_O, 30 mM l-ascorbic acid, and 4.2 mM KBr was taken as a basis, and a single parameter of interest was altered at each time. We start by varying the growth period between 3 and 20 h, as shown in Figure 3a and observe that flakes larger than 5 μm are observed already after 3 h and the average flake size increases over time, reaching a plateau around 20 h. Based on the atomic force microscopy measurements, we did not observe a direct correlation between the thickness of the flakes and the duration of growth (see Figure S7). To understand whether reactions are limited by the initial availability of Cu ions, we changed the concentration of CuSO_4_·5H_2_O between 10 and 40 mM. Figure 3b shows that the largest flake sizes can be seen at a 30 mM precursor concentration. We assume that the reaction is limited by the availability of Cu ions at low concentrations of 10–20 mM, while above 30 mM the solution is saturated and more byproducts start to form.

**Figure 3:**
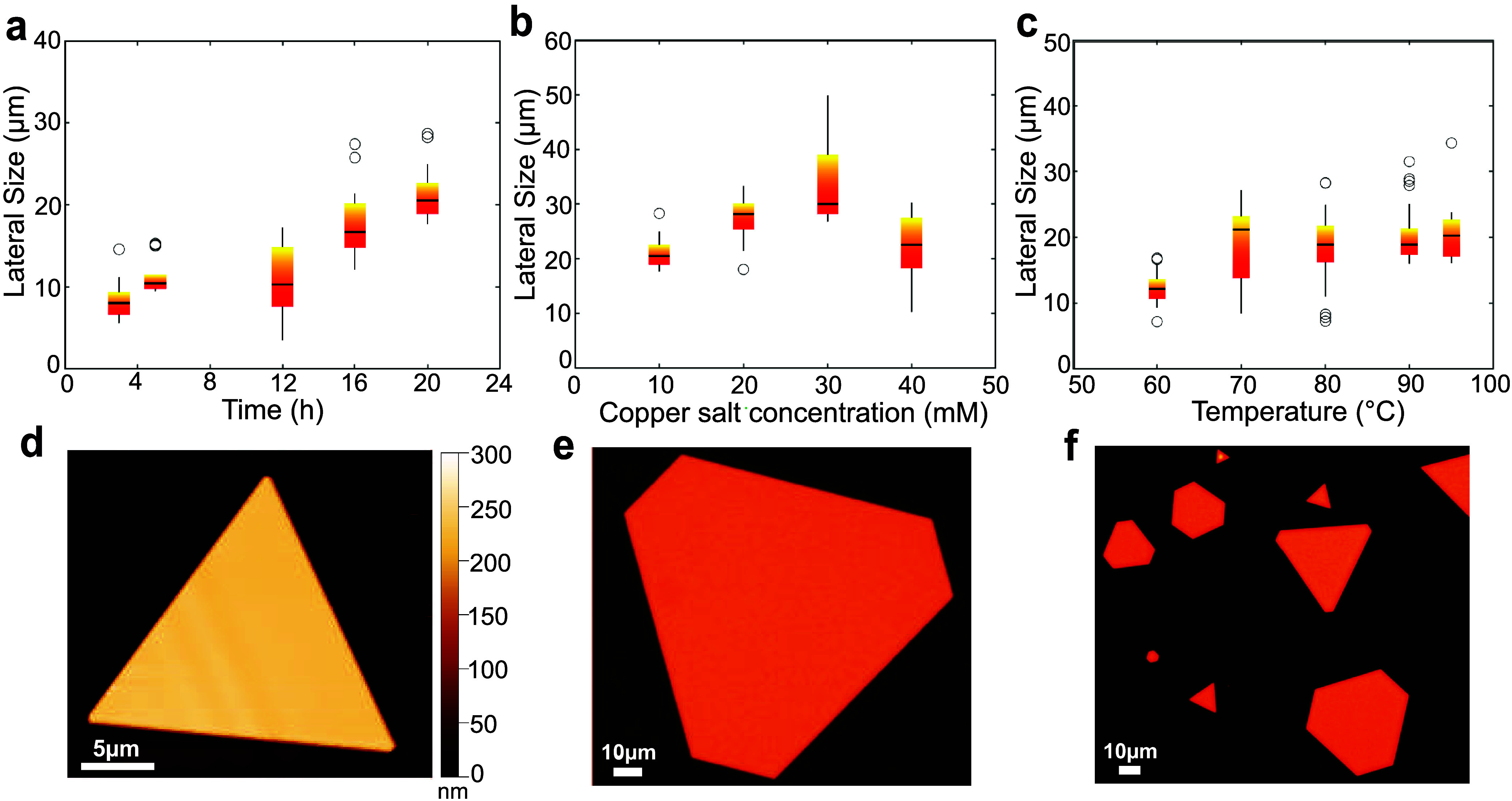
Optimization of the flake size by parametric studies. A single parameter was varied from the standard recipe at each time to acquire the trends shown in the boxplots. (a) Lateral size of the flake with respect to the growth period. (b) Influence of CuSO_4_ precursor concentration on the lateral size suggested Cu ion depletion at lower concentrations of 0–20 mM and saturation above 30 mM. (c) Temperature dependence of the lateral size in the 60–95 °C range, reaching a plateau near 90 °C. (d) AFM image of a triangular flake synthesized using the standard recipe in 20 h, showing uniform thickness of 225 nm along the flake geometry. (e) Optical micrograph of a Cu flake grown using the optimized formulation of 20 h reaction time, 95 °C, 4.2 mM KBr, 30 mM l-Ascorbic acid, and 30 mM Cu salt precursor. The hexagonal shape confirms the {111} basal plane. (f) Optical micrograph showing an increased yield of nanoparticles resulting from the recipe using optimized conditions.

At lower temperatures of 60 °C, growth predominantly occurs in the nucleation phase, producing smaller flakes, indicating that the reaction is constrained by limited kinetic energy. As shown in Figure 3c, the average lateral flake size increases at 70 °C, suggesting accelerated growth; however, a broad size distribution persists due to the presence of numerous small flakes. With further elevation of the temperature, the size dispersion decreases, reflecting more uniform growth, and the average size reaches a plateau with a few exceptionally large outliers. Since we use a water bath and deionized water as reaction medium, we remain at temperatures below 100 °C to avoid boiling of the growth solution. Alternative growth media, such as ethylene glycol, were tried, yet no noticeable formation of flakes was seen, which may be linked to the lower ion mobility in this medium.

Figure 3d shows an AFM image of a flake grown using the standard recipe that we took as a basis to perform the parametric studies. The thickness was found to be uniform (225 nm) across the entire flake surface, with a roughness mean factor of ≃180 pm, indicating a smooth top surface. Overall, variation of a single parameter at a time formed flakes with lateral sizes in the range 5 to 40 μm, as seen from Figure 3a–d. The key improvement in lateral dimensions was observed by combining the optimal conditions of the parametric studies discussed to this point, i.e., 20 h growth time, reaction temperature of 95 °C, precursor concentration of 30 mM CuSO_4_·5H_2_O, and 4.2 mM KBr. We have succeeded in developing a formulation that produces Cu microflakes with a lateral size of more than 130 μm, as shown in Figure 3e.

It is worth noting that while most of the flakes still exhibited a flat exposed surface, we noticed for the first time the formation of some flakes with steps on their top plane under these elevated conditions (see Figure S8). It is possible to avoid these features without significantly compromising on lateral size by reducing the temperature to 90 °C while maintaining the remaining parameters. However, flakes with these features can also offer an intriguing platform for energy conversion studies, as step-edge sites have been proven to be highly active catalytic sites that can reform mechanistic reaction pathways.^44–46^ These new exciting properties are unlocked thanks to our tailored surfactant-free on-substrate growth approach. Furthermore, this recipe allows an improved yield of Cu flakes with few to no undesired byproducts, as seen in Figure 3f. Additional micrographs comparing the standard and optimized recipes are included in Figure S9.

To evaluate the yield more systematically, we performed a statistical analysis of the samples prepared with different recipes using ImageJ software, the results of which are summarized in Table S1. We consider the flake yield as the number of flakes per mm^2^ as well as the surface area covered. For selectivity, we evaluate the ratio of flakes in good condition to the total structures present. All flakes with surface impurities, partially grown flakes, and flakes with step formation are considered as deformed flakes, while nonflakes refer to byproducts such as clusters and rods. The first formulation we analyzed was the combination of Cu­(NO_3_)_2_ precursor with CTAB and HMTA as organic surfactants. Despite a considerable number of 56 flakes, these were not well isolated, and the achievable size was really small with only 4 flakes larger than 10 μm. Although the surface coverage is 2.6%, it is strongly dominated by side products such as rods and clusters (see Figure S1).

In the second recipe, organic surfactants CTAB + HMTA were replaced by KBr while the Cu­(NO_3_)_2_ precursor was kept, which was selected as the ligand for this study. Although the ratio of flakes to total structures improved from 12 to 18%, this was due to a decrease in byproducts rather than an increase in the number of flakes, as can be seen in Figure S2a. This recipe gave a poor yield of only 4 flakes over a mm^2^ area, none of which were larger than 10 μm. In addition, the surface coverage is only 0.65%.

When testing different copper salt precursors with different anions, we found, as mentioned above, that the CuSO_4_ precursor provided the highest flake selectivity (summarized in Figure S2). Recipes with the other two precursors were not included in the yield analysis because it was difficult to systematically count flakes and byproducts for growth with Cu­(CH_3_COO)_2_ (Figure S2a), and there were no flakes but only prisms for growth with CuCl_2_ (Figure S2b).

It can be seen that the precursor is a key factor for product selectivity, with flake selectivity increasing from 18 to 51.9% when the precursor is changed from Cu­(NO_3_)_2_ to CuSO_4_. The combination of CuSO_4_ and KBr, which we refer to as the standard formulation in this study, already gave a significantly high selectivity for the flakes. However, the covered area was small: only 0.26% of the surface was covered with flakes, and only 17% of the flakes were larger than 10 μm. These figures indicate that although the nucleation was successful and many well-isolated flakes were formed, the growth phase was incomplete. This can also be seen from the micrographs in Figure S9a,c, where the predominant product is flakes, but these are only of a small average size.

In the optimized recipe, which was the first and only one to form structures with dimensions greater than 100 μm, not only the average size but also the selectivity toward flakes, yield, and surface coverage were significantly improved, as shown in Figure S9d–f. The surface coverage increased from 0.264 to 5%, a 20-fold increase over the standard recipe, while the selectivity increased from 51.9 to 73.26%. This suggests that the growth phase could be sustained for a greater number of structures upon use of the optimized recipe. In addition, 35 of the 44 flakes were larger than 10 μm, corresponding to a ratio of 79%. For each batch, two borosilicate substrates of 24 × 24 mm^2^ are available on which the flakes can grow on both the outer and gap surfaces, resulting in a total surface area of ≈2300 mm^2^. Even with possible fluctuations in the yield along the surface, it can be expected that several hundred flakes will grow in a single experiment. Overall, this statistical analysis shows that the optimization was successful in promoting growth toward better values such as selectivity, isolation of flakes, and yield. We see this as a major advantage that facilitates the integration of devices and systematic studies on this material platform.

## Conclusions

We report here a highly repeatable, straightforward approach to synthesize exceptionally large-area Cu microflakes on borosilicate glass substrates. By precisely tuning the halide type and concentration, we explored the powerful capabilities of bromide ions to control the flake morphology and, quite remarkably, to enhance the flake stability by mitigating surface oxidation. At optimized conditions, we achieve high-yield anisotropic growth, with an aspect ratio of up to 400 and lateral sizes over 130 μm, at least 2 orders of magnitude higher than in previous studies. By overcoming the size limitations of previous synthesis methods, our results open new possibilities for using monocrystalline Cu flakes in fundamental research, photonics, and catalytic applications.^7,47^

We perform various compositional analysis techniques such as SEM-EDS, STEM-EDS, EELS, and XRD, which reveal the monocrystalline, fcc metallic structure of the Cu flakes. Copper is the most widely studied material for catalysis and energy conversion devices, given its unique ability to support the formation of multicarbon products on its surface. These large-area, surfactant-free Cu flakes with a well-defined (111) top surface provide a promising platform for catalysis, allowing controlled studies on selectivity, activity, and complex reaction mechanisms.^48^ In addition, we envision that these flakes will be highly beneficial for plasmonic and nanophotonic devices thanks to their single crystallinity.^20^

## Supplementary Material



## Data Availability

The data underlying this manuscript will be available at Zenodo.

## Notes

Growth in the presence of CTAB and HMTA; influence of Cu salt precursor on flake growth; comparison of colloidal grown versus substrate grown flakes; SEM-EDS analysis of flakes and nuclei; flake stability; electron energy loss spectroscopy (EELS) mapping; optical characterization of Cu flakes; analysis of flake thickness with respect to growth period; step formation on flakes at high temperatures; statistical analysis of flake yield for different recipes; comparison of yield between the standard and optimized recipes via micrographs (PDF)
