# Supplementary material for: Large-Area Monocrystalline Copper Microflake Synthesis
Source: J Phys Chem C Nanomater Interfaces. 2025 Jun 16;129(25):11574–82. doi: 10.1021/acs.jpcc.5c00654 (PMC12207664; doi:10.1021/acs.jpcc.5c00654)
Supplement: Supplementary file 1 [file jp5c00654_si_001.pdf]

Large area monocrystalline copper microflake synthesis

Elif Nur Dayi, Diotime Pellet, Priscila Vensaus, Fatemeh Kiani, Alan R. Bowman, Omer Can Karaman, and Giulia Tagliabue*

Laboratory of Nanoscience for Energy Technologies (LNET), STI,
École Polytechnique Fédérale de Lausanne, 1015 Lausanne, Switzerland

E-mail: giulia.tagliabue@epfl.ch

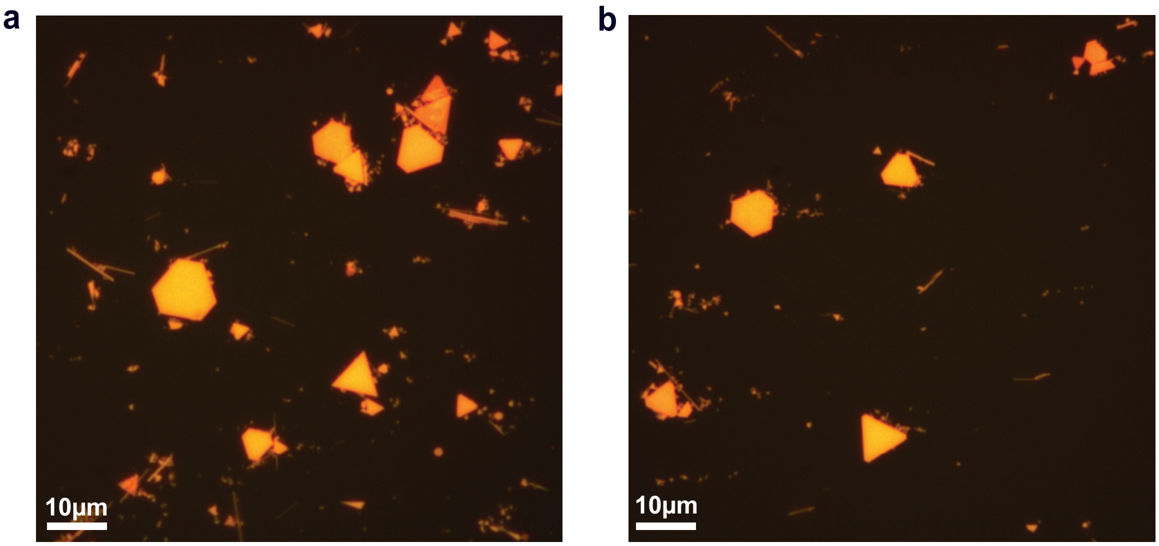

Figure S1: Cu flake growth on borosilicate substrates using copper nitrate trihydrate ($Cu(NO_3)_2 \cdot 3H_2O$, 50 mg) as the salt precursor, with L-Ascorbic acid (100 mg) as the reducing agent and hexadecyltrimethylammonium bromide (CTAB, 100 mg) and hexamethylenetetramine (HMTA, 100 mg) as surfactants. The recipe was adapted from Luc et al[1]. The flakes were not isolated and were surrounded by numerous side products such as nanorods.

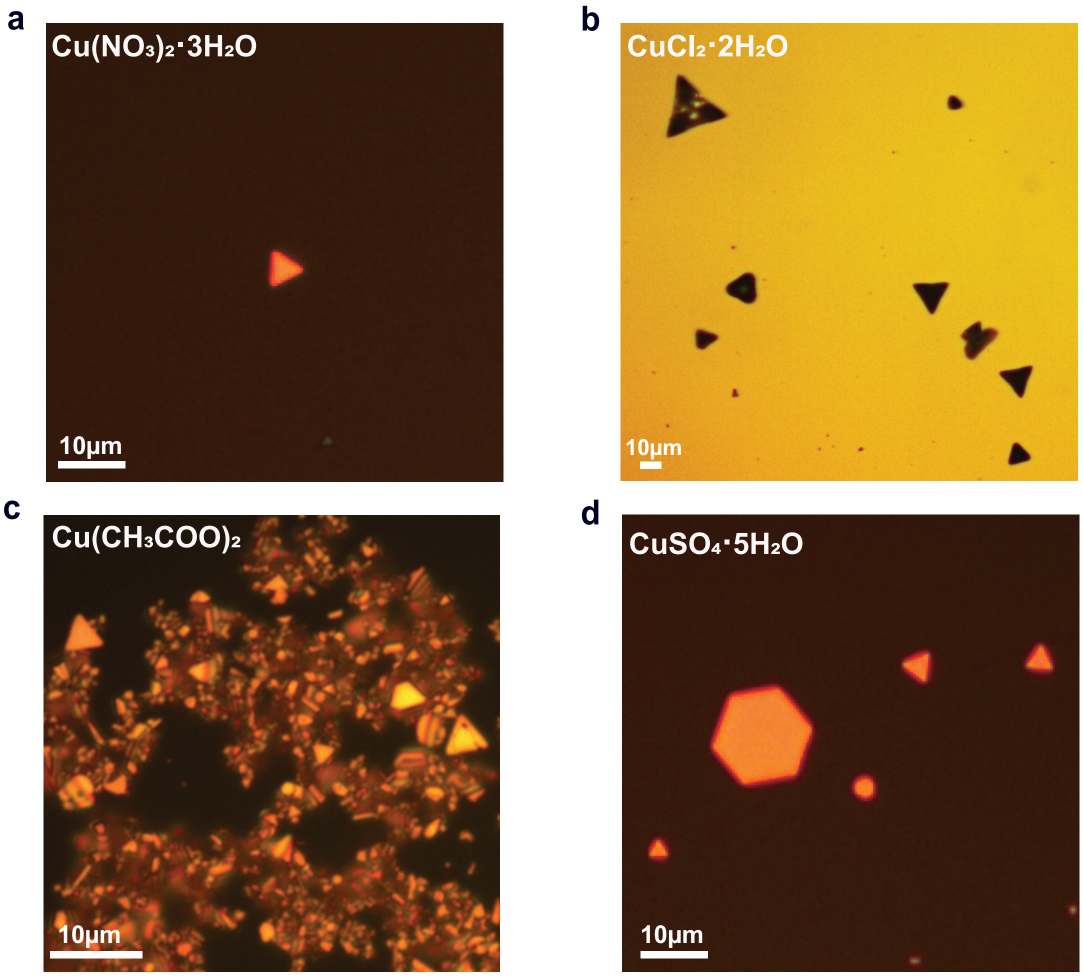

Figure S2: Dependence of Cu flake growth on the Cu salt precursor. **a** Copper nitrate trihydrate generated flakes with limited size. **b** Copper chloride dihydrate led to thick prism-like structures and no flakes with smooth surfaces. **c** Copper acetate resulted in small flakes with lateral sizes around 5 μm. **d** Copper sulfate pentahydrate, the chosen precursor for this study, yielded copper flakes of greater number and larger sizes.

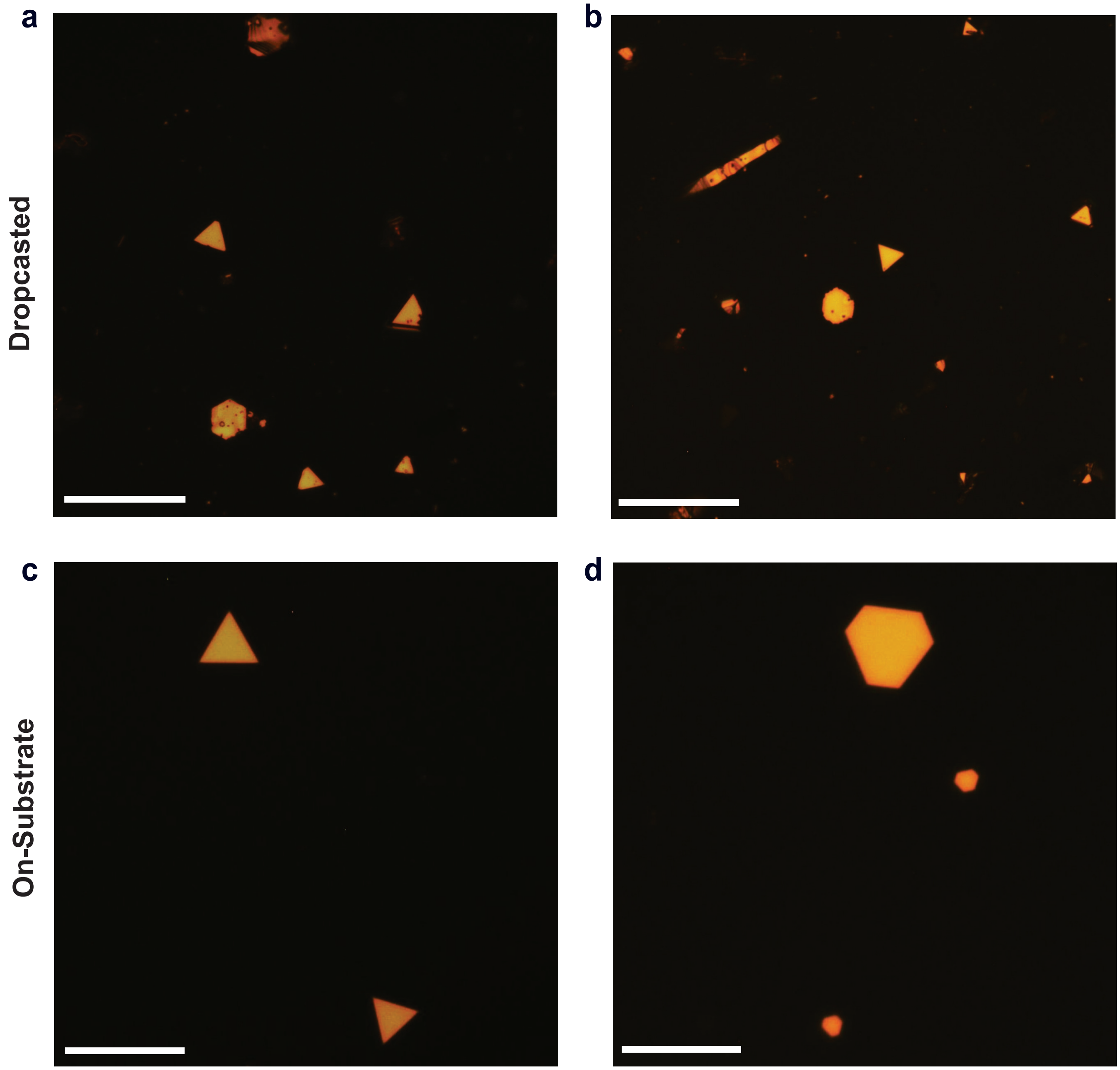

Figure S3: Comparison of colloidal grown dropcasted flakes with on-substrate grown flakes. All scale bars indicate 50 μm. **a-b** Bright-light images showing products grown in solution. Solution collected immediately after reaction was centrifuged in pure ethanol and deionized water with 1:1 ratio for 3 cycles and the final solution was dropcasted onto a clean borosilicate substrate at room temperature, followed by drying under ambient conditions. The flakes tend to exhibit structural deformations, and by-products such as rods and clusters form in growth solution compared to flakes grown directly on substrates. **c-d** Bright-light images of flakes directly grown on borosilicate glass substrates for the same synthesis. The flakes are well isolated from any side-products and less likely to have structural deformations.

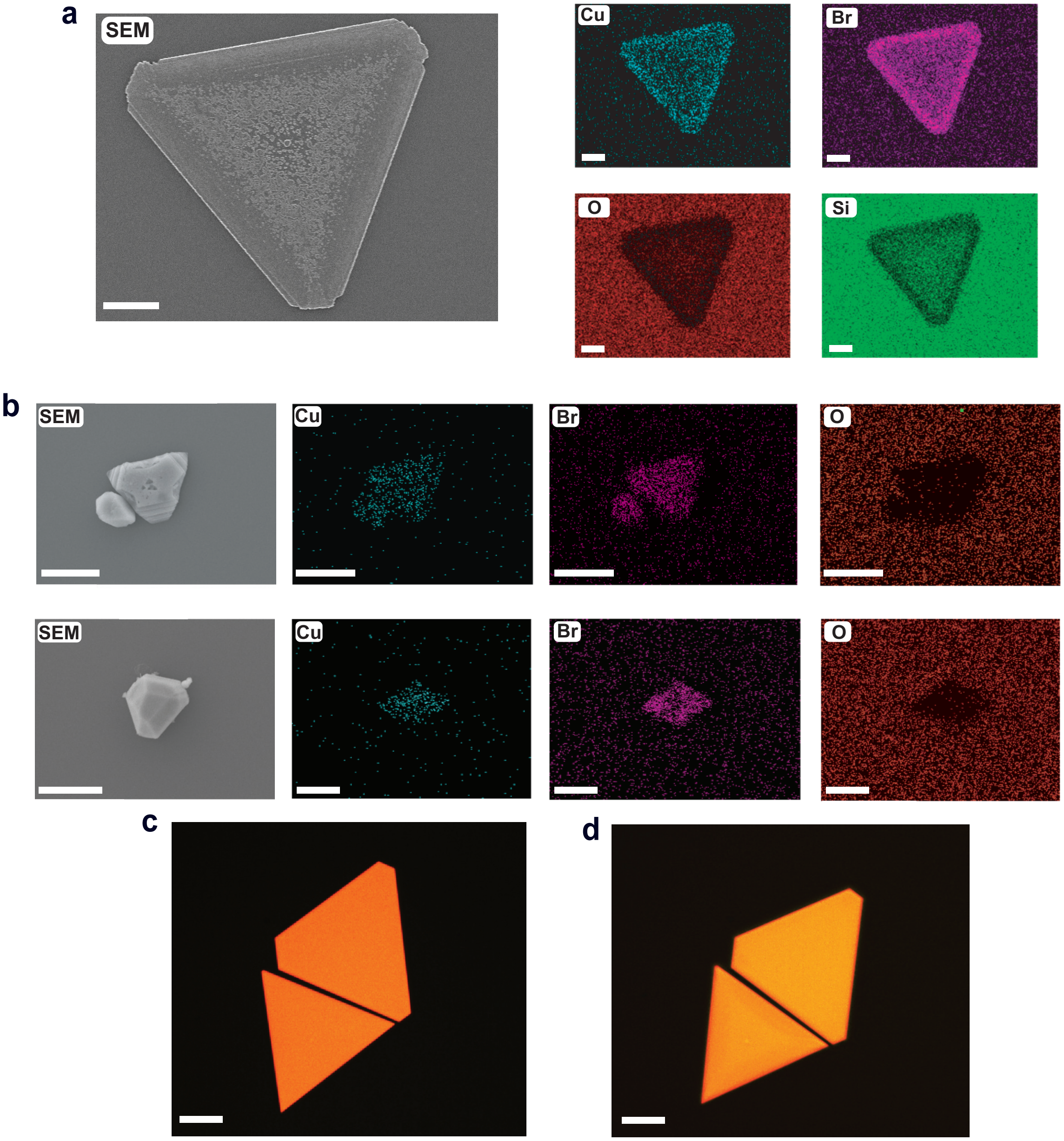

Figure S4: SEM-EDS elemental analysis and stability of Cu flakes. All scale bars indicate 5 μm. **a** SEM image of a copper flake synthesized using high concentration of KBr (10.2 mM), showing etching at the center of the flake and the corresponding EDS maps for Cu(blue), Br(pink), O(red), Si(green). The following lines were used to construct these images: Cu Lα1-2, O Kα1, Si Mα1 and Br Lα1-2. A continuous bromide layer around the edges of the flake is visible. The faint copper signal and the increased signal for Si and O, which come from the underlying glass substrate suggest thinning down of the flake at the center due to etching effects at high bromide concentration. **b** SEM micrographs of CuBr particles and the corresponding elemental maps of Cu, Br and O. **c** Optical micrograph of two flakes right after synthesis, prepared with 4.2 mM KBr, which is referred to as the standard recipe in this study. **d** Optical micrograph of the same flakes a month after synthesis, stored under ambient conditions.

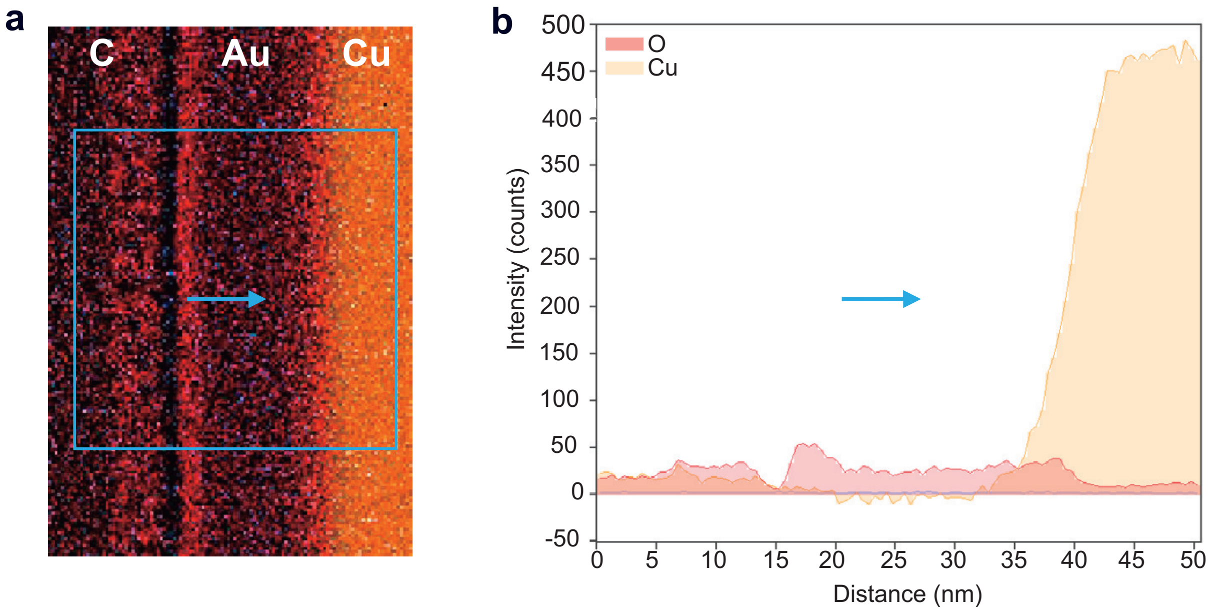

Figure S5: Electron energy loss spectroscopy (EELS) analysis on the cross-sectional Cu flake lamella. **a** EELS map where the blue frame marks the area of interest for the spectrum in b. The blue arrow inside indicates direction of EELS profile. **b** Corresponding EELS spectrum. O is detected as noise, while a strong Cu signal from the flake, confirming the metallic nature of the Cu flake.

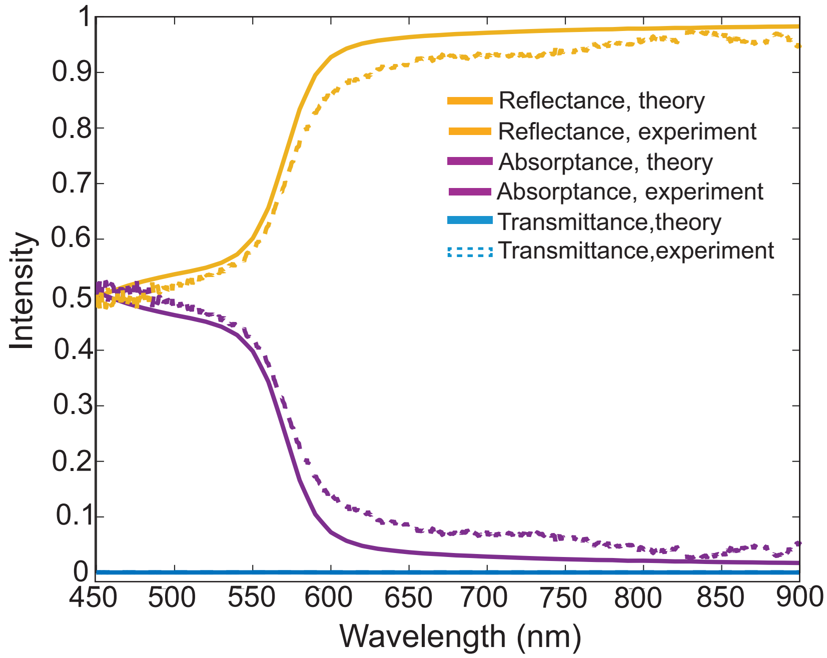

Figure S6: Microscale absorptance measurements performed on a Cu flake. Experimental (dashed) and theoretical (solid) values for reflectance (orange), transmittance (blue) and absorptance (purple) were compared. Theoretical values were calculated using transfer matrix method for a Cu film of 200 nm thickness using optical parameters of Cu from McPeak.[2]. As expected from metallic Cu, high reflectance and low transmittance were observed across the visible spectrum.

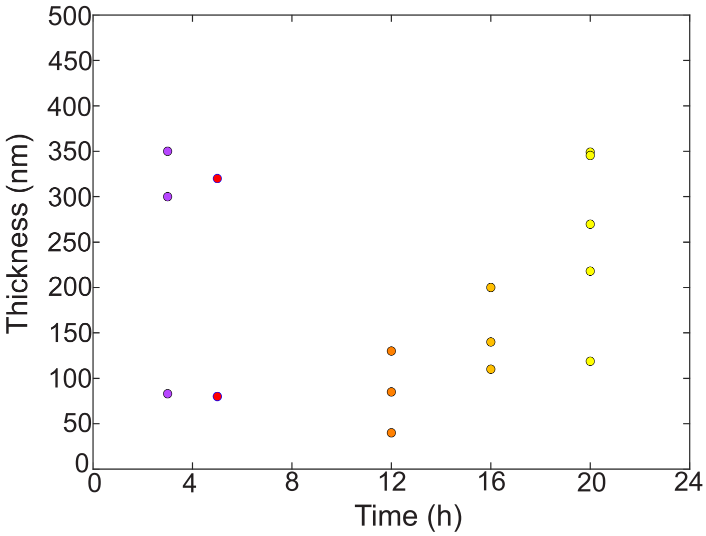

Figure S7: Flake thickness measured by atomic force microscopy for samples grown for different growth period. No significant correlation between the flake thickness and growth time was observed, and similar thickness range was observed for the shortest and the longest durations.

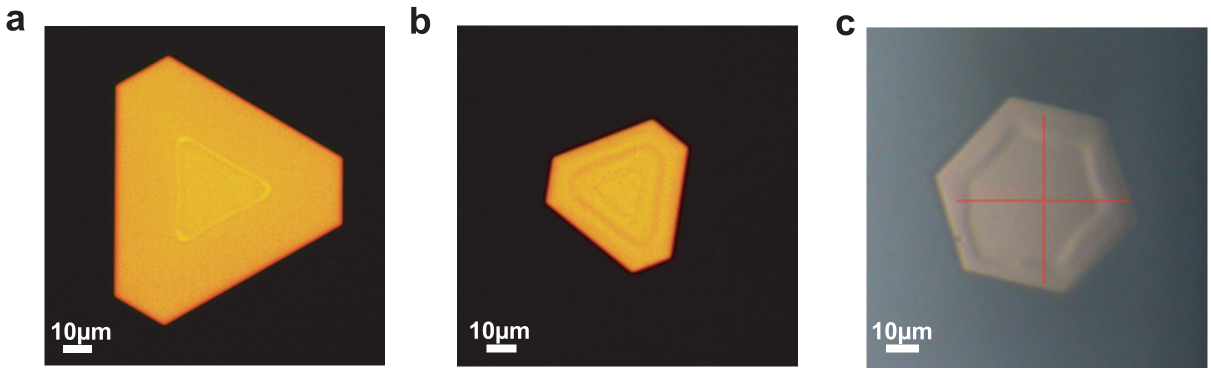

Figure S8: Step formation on the top surface of some flakes synthesized at 95°C. **a** Optical micrograph of a truncated hexagonal flake with a triangular step. **b** Optical micrograph of a flake with multiple steps. **c** Microscopy image recorded with the digital camera of the atomic force microscope, showing a hexagonal flake with edge features.

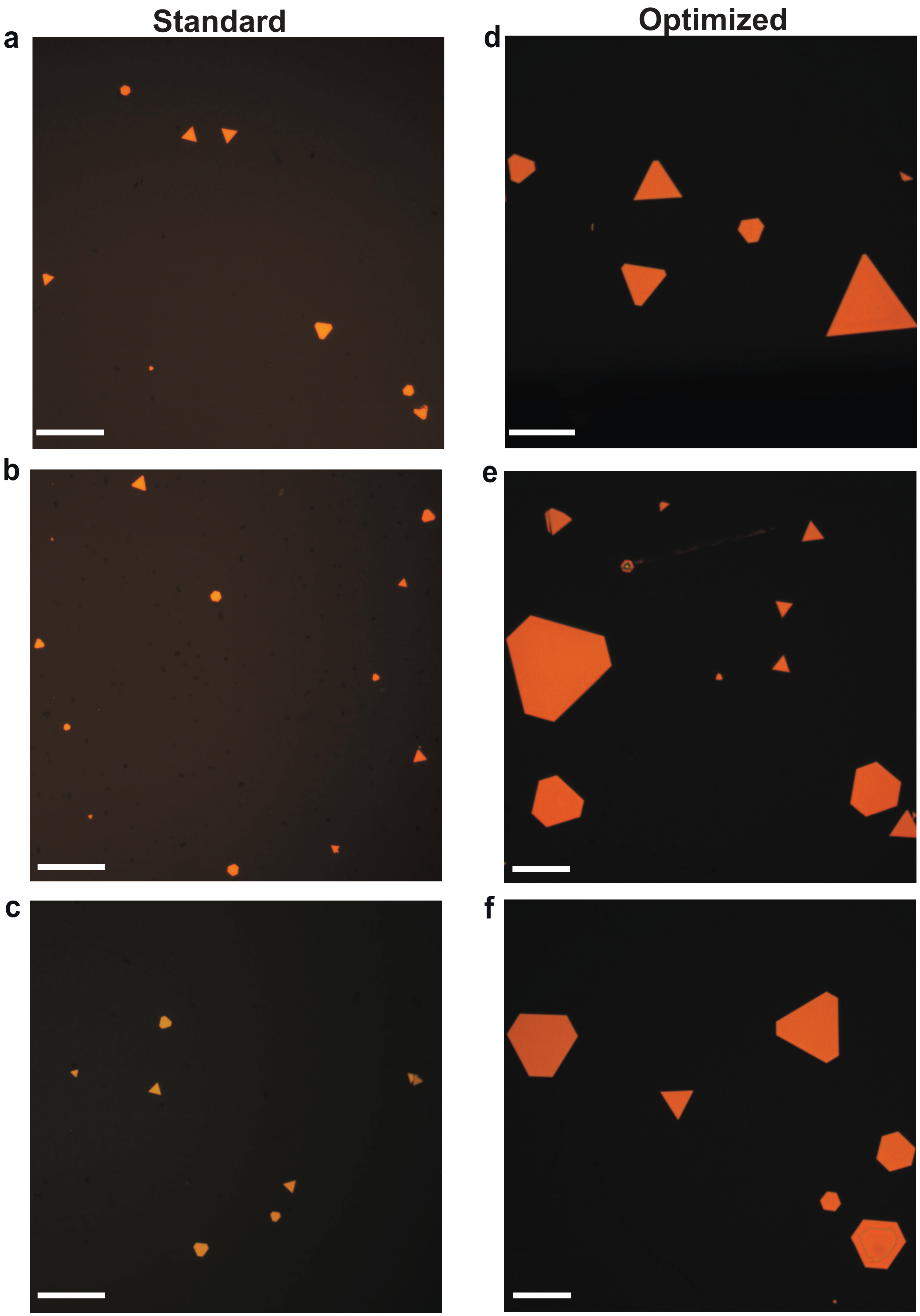

Figure S9: Bright-light images of flakes grown with standard (a-c) and optimized recipe (d-f). All scale bars indicate 50 μm.

Table S1: Analysis of flake yield for different recipes. We consider the flake yield as the number of flakes per mm^2 as well as the surface area covered. For selectivity, we evaluate the ratio of flakes in good condition to the total structures present. All flakes with surface impurities, partially grown flakes, and those with step formation are considered deformed flakes, while non-flakes refer to by-products such as clusters, rods, etc.

**Recipe Name**	**Reaction Conditions**	**Flake** (num/mm^2)	**Size >10μm** (num/mm^2)	**Deformed Flakes** (num/mm^2)	**Non-flake** (num/mm^2)	**Total Structures** (num/mm^2)	**Covered Area** (%)	**Healthy Flakes to Total** (%)
$CuNO_3$ +CTAB +HMTA	10mM Cu precursor<br>30mM L-Asc. acid<br>14mM CTAB<br>36mM HMTA<br>80°C<br>20h	56	4	62	318	436	2.6	12.84
$CuNO_3$ +KBr	10mM Cu precursor<br>30mM L-Asc. acid<br>4.2mM KBr<br>80°C<br>20h	4	0	8	10	22	0.65	18
$CuSO_4$ +KBr (standard)	10mM Cu precursor<br>30mM L-Asc. acid<br>4.2mM KBr<br>80°C<br>20h	51	9	24	23	98	0.264	51.98
$CuSO_4$ +KBr (optimized)	30mM Cu precursor<br>30mM L-Asc. acid<br>4.2mM KBr<br>95°C<br>20h	44	35	12	5	60	5.120	73.26

References

(1) Luc, W.; Fu, X.; Shi, J.; Lv, J.-J.; Jouny, M.; Ko, B. H.; Xu, Y.; Tu, Q.; Hu, X.; Wu, J. et al. Two-dimensional copper nanosheets for electrochemical reduction of carbon monoxide to acetate. *Nat. Catal.* **2019**, *2*, 423–430.

(2) McPeak, K. M.; Jayanti, S. V.; Kress, S. J. P.; Stefan Meyer; Iotti, S.; Rossinelli, A.; Norris, D. J. Plasmonic films can easily be better: Rules and recipes. *ACS Photonics* **2015**, *2*, 326–333.
